# Electroacupuncture Enhances Neuroplasticity by Regulating the Orexin A-Mediated cAMP/PKA/CREB Signaling Pathway in Senescence-Accelerated Mouse Prone 8 (SAMP8) Mice

**DOI:** 10.1155/2022/8694462

**Published:** 2022-02-04

**Authors:** Zhitao Hou, Xinyu Yang, Yang Li, Jing Chen, Hongcai Shang

**Affiliations:** ^1^Key Laboratory of Chinese Internal Medicine of the Ministry of Education, Dongzhimen Hospital Affiliated to Beijing University of Chinese Medicine, Beijing 100700, China; ^2^College of Basic Medical and Sciences, Heilongjiang University of Chinese Medicine, Harbin, 150040 Heilongjiang, China; ^3^Fangshan Hospital Beijing University of Chinese Medicine, Beijing 102400, China

## Abstract

Learning and memory disorders and decreased neuroplasticity are the main clinical manifestations of age-induced cognitive dysfunction. Orexin A (OxA) has been reported to show abnormally elevated expression in the cerebrospinal fluid (CSF) of patients with Alzheimer's disease (AD) and to be associated with cognitive impairment. Here, we further assessed whether the excitatory neurotransmitter OxA is involved in neuroplasticity and cognitive function in senescence-accelerated mouse prone 8 (SAMP8) mice. In this study, we investigated the mechanism of OxA by using behavioral tests, CSF microdialysis, immunofluorescence, toluidine blue staining, gene silencing, transmission electron microscopy, and Western blotting. The results showed that 10 Hz electroacupuncture (EA) effectively alleviated learning and memory impairment in 7-month-old SAMP8 mice, reduced OxA levels in the CSF, increased the level of the neurotransmitter glutamate, alleviated pathological damage to hippocampal tissue, improved the synaptic structure, enhanced synaptic transmission, and regulated the expression of cAMP/PKA/CREB signaling pathway-related proteins. These results suggest that EA enhances neuroplasticity in SAMP8 mice by regulating the OxA-mediated cAMP/PKA/CREB signaling pathway, thus improving cognitive function. These findings suggest that EA may be beneficial for the prevention and treatment of age-induced cognitive impairment.

## 1. Introduction

Cognitive impairment is a neurodegenerative disease that seriously affects the health of elderly individuals [1]. According to the Alzheimer's Association's latest annual report for 2021 [2], the number of people over 65 with dementia is expected to reach 12.7 million by 2050. We propose that age-related degeneration of synapses may be an important focus for elucidating the mechanism of Alzheimer's disease (AD) because synapses are the cellular basis for the information process, memory formation, movement, sensations, emotions, and skills.

Orexin A (OxA)/hypocretin 1 is an excitatory neuropeptide that is synthesized by neurons located in the lateral hypothalamus, distributed in most brain regions, including the hippocampus, and plays an important role in the initiation and maintenance of wakefulness [3]. The OxA receptor OX1R/HCRT1R is a G protein-coupled receptor involved in a variety of physiological functions, including learning and memory [4]. Clinical studies have reported that patients with increased levels of OxA in the cerebrospinal fluid (CSF) often present with circadian rhythm disturbances [5, 6], decreased synaptic plasticity [7, 8], and neurotransmitter transport dysfunction [9, 10], as well as changes in the expression of AD-related biomarkers, such as increased levels of phosphorylated Tau and A*β* in the CSF [11, 12]. As the disease progresses, patients with AD with abnormally elevated OxA levels always exhibit a significantly poorer mental status and lower cognition scores [13]. These findings seem to reveal a strong correlation between OxA levels and cognitive impairment.

Electroacupuncture (EA) is a common nondrug therapy that is currently widely used in the clinical treatment of cognitive impairment in Northeast Asia [14]. Our previous study found that EA significantly improves the learning and memory ability of patients with cognitive impairment and can reduce the level of OxA in patients [15]. Administration of suvorexant, a commonly used orexin receptor antagonist, improves cognitive function, promotes cAMP-responsive element-binding protein (CREB) phosphorylation, and reduces A*β* accumulation in 9-month-old APP/PS1 mice [16]. However, relevant studies have not confirmed whether EA affects learning and memory deficits induced by neurodegenerative changes resulting from senescence through OxA or whether it has a regulatory effect on synaptic plasticity mediated by phosphorylation of cAMP/PKA/CREB-related proteins. Considering these previous findings, we hope to determine the effect of EA in alleviating cognitive dysfunction in a mouse model of aging-induced cognitive impairment and explore the underlying mechanism.

## 2. Materials and Methods

### 2.1. Animals and Treatment

All animal experiments were conducted in accordance with the guidelines of the Animal Experiment Ethics Committee of Heilongjiang University of Chinese Medicine for the use of experimental animals and the experimental animal use guidelines and specifications of Heilongjiang University of Chinese Medicine. Seven-month-old senescence-accelerated mouse prone 8 (SAMP8) mice and senescence-accelerated mouse resistant 1 (SAMR1) mice (purchased from the Animal Experiment Center of The First Affiliated Hospital of Tianjin University of Traditional Chinese Medicine (Experimental Animal Production License no. SCXK (Tianjin)2015-0003) were housed at a controlled temperature (20–22°C) and humidity (50%–70%) on a 12-hour light/dark (LD) cycle (lights on at 07:00 and lights off at 19:00) and provided free access to food and water. The zeitgeber time (ZT) system was used for the LD cycle, with the lights being turned on at ZT0 (07:00) and the lights being turned off at ZT12 (19:00).

After 7 days of adaptive feeding, 32 cognitively impaired SAMP8 mice and 8 SAMR1 mice with normal cognition were used for the Morris water maze (MWM) test. The SAMP8 mice and SAMR1 mice were divided into 4 groups: the vehicle-treated SAMP8 (model) group (*n* = 16), 10 Hz EA-treated SAMP8 (10 Hz EA) group (*n* = 8), 10 Hz nonacupoint EA-treated SAMP8 (10 Hz nac-EA) group (*n* = 8), and vehicle-treated SAMR1 (control) group (*n* = 8).

**Figure 1:**
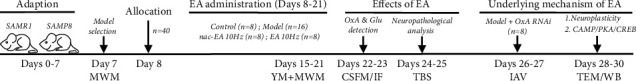
Experimental protocol. MWM: Morris water maze; YM: Y maze; nac: nonacupoint; CSFM: cerebrospinal fluid microdialysis; IF: immunofluorescence; TBS: toluidine blue staining; IAV: injection of adenovirus vector; TEM: transmission electron microscope; WB: Western blotting.

The operation method of electroacupuncture is based on the method adopted by similar high-level research [17, 18], combined with our previous studies [15, 19] and preliminary experimental exploration of this research, and finally established the most suitable method for this study. The details are as follows: for EA, 0.18 × 13 mm stainless steel needles (Guizhou And Medical Appliances Ltd., Guizhou, China) were inserted at the GV20 and bilateral ST36 acupoints. A needle was inserted at the GV20 acupoint, which is located in the middle of the mouse parietal bone and the midpoint of the two ear tips, downward at a 15 degree angle to a depth of 3 mm. A needle was inserted at the ST36 acupoint, which is located 2 mm lateral to the anterior tibial nodule and 3 mm below the knee joint, vertically downward to a depth of 3 mm. Electrical stimulation with an intensity of 1 mA and a frequency of 10 Hz was administered using a HANS-200A acupoint nerve stimulator (Nanjing, China) once a day for 14 consecutive days for 30 min each time. In the nac-EA 10 Hz group, needles were inserted at 3 nonacupoint sites at the midpoint of the tail and 1 cm to each side to a depth of 3 mm. These sites did not correspond to any acupoints, but electrical stimulation was administered as described above. The control and model groups did not receive EA treatment. After confirming the effect of 10 Hz EA treatment in improving the learning and memory of SAMP8 mice and reducing the level of OxA in the CSF, 16 mice in the model group were randomly divided into 2 groups, the SAMP8-OxA-RNAi (OxA RNAi) group (*n* = 8), which underwent gene silencing, and the model group, to determine the mechanism of EA treatment. The experimental process is shown in Figure 1.

### 2.2. Behavioral Evaluation

#### 2.2.1. Y Maze Spontaneous Alternation Test

To assess spatial learning and memory, each mouse underwent the Y maze test. The Y maze used in this experiment was composed of three arms with an incline angle of 120° between them. Each arm was 30 cm long, 6 cm wide, and 15 cm high, and the arms were connected by a movable partition in the central region. The three arms of the Y maze were randomly assigned as the novel arm, initial arm, and other arm. In the training phase, the initial arm and other arm of the Y maze were opened, and 30 minutes after EA treatment, the mice were placed in the initial arm or other arm of the maze and allowed to freely explore the initial arm and other arm for 10 min. After the training phase, the mice were placed back in their original cages and subjected to a test phase one hour later. In the test phase, the novel arm, initial arm, and other arm of the Y maze were opened. The mice were placed in the initial arm facing the wall and allowed to freely explore the three arms for 8 min. The experiment was carried out in a quiet and dim environment. Video tracking was used throughout the experiment. The test equipment was cleaned with 75% ethanol at the end of the training and testing phases to eliminate any odor traces. Entry into the three different arms in turn was considered a correct alternation, and the spontaneous alternation rate (%) was calculated as the number of correct alternations/(the total number of arm entries − 2) × 100%.

#### 2.2.2. MWM Test

The MWM consisted of a large circular plastic swimming pool (100 cm in diameter and 50 cm high) filled with water with a temperature of 22–24°C to a depth of 32 cm. A small escape platform (12 cm in diameter) was placed 1 cm below the surface of the water in the first quadrant. Visual cues of different shapes were placed on the walls of the pool. The mice were trained on the water maze for five days. On day 6, the escape platform was removed and the mice were tested. The cutoff time for each test was 90 s. If a mouse did not reach the platform within 90 s, it was gently guided to the platform and kept there for 20 s to observe its surroundings. During the test, the swim path, escape latency, time spent in the target quadrant, and swimming speed were recorded and analyzed using SuperMaze 3.3.0.0 software (Shanghai New Maze Information Technology Co. Ltd., Shanghai, China).

### 2.3. Analysis of Neurotransmitter Levels

We measured the levels of two neurotransmitters, namely, OxA and glutamate, in the CSF. CSF microdialysis was used to obtain CSF, and then, the levels of the two neurotransmitters in the CSF were measured using an ELISA kit. CSF microdialysis was performed as follows: on the second day after cannula implantation, a probe was inserted into each cannula, vests were put on the mice, and the mice were placed in a mouse autonomous activity device. Microsyringes and low-temperature collectors were connected to both sides of the probe, and 90 *μ*L CSF was collected at each of 8 time points, i.e., ZT3, ZT6, ZT9, ZT12, ZT15, ZT18, ZT21, and ZT24, at a rate of 30 *μ*L per hour for ELISA. The OxA (MM-0302R) and glutamate (MM-0601R) kits used in this experiment were obtained from Jiangsu Enzyme Industry Co. Ltd.

### 2.4. Immunofluorescence Analysis

A wax block was fixed to the clamping table of a paraffin slicer, and 4 *μ*m thick tissue sections were cut. The tissues were dewaxed, hydrated in gradient alcohol solutions, and washed with PBS 3 times for 5 min each. An appropriate amount of citric acid repair solution (pH 6.0) was added to the repair box, the slices were placed in the repair box, and the box was sealed with adhesive tape to prevent the repair solution from boiling over. The repair box was placed in a microwave oven, heated for 8 min at medium heat, kept warm for 8 min, and heated for 7 min at medium–low heat. The slices were cooled naturally after repair. After being washed with PBS 3 times for 5 min each, the tissues were evenly covered with 10% normal rabbit serum and blocked at room temperature for 30 min. The tissue was evenly covered with diluted primary antibody and incubated overnight at 4°C. The slices were removed from the refrigerator, rewarmed at room temperature for 30 min, and washed with PBS 3 times for 5 min each. Then, the tissue was completely covered with fluorescent secondary antibodies against orexin and GFAP (Cell Signaling Technology, Danvers, MA) and incubated at room temperature in the dark for 60 min. The secondary antibody was rinsed off, and the sections were placed on a rack and washed with PBS 3 times for 5 min each. The tissue was completely covered with DAPI for 8 min to stain the nuclei and then washed with PBS 3 times for 5 min each. The liquid around the tissue was removed, half a drop of antifluorescence quenching agent was added with a rubber dropper, and the slide was covered with a glass plate. Fluorescence microscopy (DS-F12; Nikon, Tokyo, Japan) was used to observe the staining and image the tissues.

### 2.5. OxA-RNAi Adenovirus Vector Transfection by Injection

One of three constructs was selected for transfection, and the gene sequence of the selected adenovirus was as follows: GCGGCCGCACTAAAGATATTTGCATGTCGCTATGTGTTCTGGGAAATCACCATAAACGTGAAATGTCTTTGGATTTGGGAATCTTATAAGTTCTGTATGAGACCACTCGTCTCTACGAACTGTTGCACGttcaagagaCGTGCAACAGTTCGTAGAGACtttttTCTAGA. Stereotactic injection was performed as follows: before injection, the mice were deeply anesthetized by intraperitoneal injection 80 mg/kg body weight 2% pentobarbital sodium in saline. The hair on the head and back of each mouse was trimmed with scissors, the skin on the mouse head was disinfected with 75% alcohol and dried with a cotton ball, and an incision was made in the skin above the skull using bregma as a reference point. The mice were fixed to a stereotaxic instrument with ear bars. The Franklin and Paxinos mouse brain atlas [20, 21] was used to determine the appropriate coordinates (−2.79 A/P, 1.75 M/L, and 2.5 D/V, that is, 2.79 mm posterior to bregma, 1.75 mm lateral on either side of the midline, and 2.5 mm deep from the skull surface). A 2 mm hole was carefully drilled on the skull at the implantation site using an animal skull drill at a slow and uniform speed to avoid damage to the subcranial cortex, which may cause bleeding or brain tissue damage. The stereotaxic arm was slowly moved to the appropriate location, and a microsyringe with a needle was used to slowly inject 1 *μ*L adenovirus at a rate of 0.25 *μ*L/min over 4 min. The microsyringe was kept in place for 2 min after the injection and then slowly removed. The skull was sealed with a small amount of bone wax, the incision was sutured, and routine disinfection was performed.

### 2.6. Neuropathological Staining

Hippocampal tissue was fixed with 4% paraformaldehyde, dehydrated, and embedded in paraffin. The specimens were cut into 6 *μ*m thick slices and stained with toluidine blue. Pathologists observed the pathological changes in the hippocampus after toluidine blue staining under 200, 400, and 800x magnification. Finally, changes in pyramidal cells in the CA1 and CA3 regions of the hippocampus and Nissl bodies were observed.

### 2.7. Western Blotting

Mice from each group were decapitated at ZT3, and their brains were collected. Hippocampal tissues were isolated; weighed; successively treated with lysis buffer (10 mL/g), a broad spectrum phosphatase inhibitor (1 : 100) and PMSF (1 : 100); and ultrasonicated on ice. The supernatant was stored at 4°C for 4 h and centrifuged at 13000 r/min for 15 min, and the protein concentration was determined by the BCA assay. Thirty micrograms of protein was heated for 5 min, separated by SDS-PAGE, and transferred onto a polyvinylidene fluoride (PVDF) membrane, which was incubated with an anti-rabbit anti-polyclonal antibody (1 : 500) or mouse anti-*β*-actin antibody (1 : 500) overnight at 4°C. Then, the membrane was incubated with an HRP-labeled goat anti-rabbit IgG (1 : 5000) or HRP-labeled goat anti-mouse IgG (1 : 5000) secondary antibody at 4°C for 2 h and ECL reagent was added for development. ImageJ software was used to analyze the levels of OxA, cAMP, PKA, CREB, phosphorylated CREB (pCREB), GluN1, GluN2A, GluA2, synaptophysin (SYP), and postsynaptic density protein-95 (PSD95) (all antibodies are purchased from Cell Signaling Technology, Danvers, MA).

### 2.8. Transmission Electron Microscopy (TEM)

Hippocampal tissues were isolated, and 1 mm^3^ tissue blocks were cut from the area containing the CA1 region and placed in 0.01 m PBS containing 2.5% glutaraldehyde for at least 2 h. The tissues were then fixed in 1% osmium for 2 h at 4°C and washed twice with PBS for 5 min each. The tissues were dehydrated in graded acetone solutions and immersed in acetone embedding solution (1 : 1) for 2 h at 35~37°C. Then, they were embedded in epoxy resin 812 for 2 h at 35~37°C and incubated in 2% uranium acetate aqueous solution for 2 h. The samples were placed on an embedding plate (a special porous rubber template), placed in an embedding agent, and incubated in an oven at 35°C for 12 h, 45°C for 12 h, and 60°C for 24 h to allow polymerization. Then, the tissue blocks were cooled naturally to increase their hardness. An LKB ultrathin slicing machine (slice thickness of 10–100 nm) was used to cut the tissues, and the slices were stained with uranium dioxane acetate and lead citrate for 15–30 min, immediately rinsed with ddH_2_O, differentiated with 0.02 mol sodium hydroxide, rinsed with ddH_2_O, and dried. The ultrastructure of the mitochondria, synapses, gap junctions, and myelin sheaths in the hippocampal CA1 region were observed by TEM after drying.

### 2.9. Statistical Analysis

All data are presented as the mean ± standard error and were analyzed by SPSS 22.0 and GraphPad 9.2.0 software. Escape latency from the MWM test was analyzed using three-way repeated measures analysis of variance (ANOVA). Two-way ANOVA and 2-sample *t*-tests were used to analyze the ELISA and Western blot data. Additional data were compared using 2-way ANOVA and post hoc analysis. *P* < 0.05 was considered statistically significant.

## 3. Results

### 3.1. EA at 10 Hz Alleviates Learning and Memory Deficits in SAMP8 Mice, as Evaluated by the Y Maze Test

**Figure 2:**
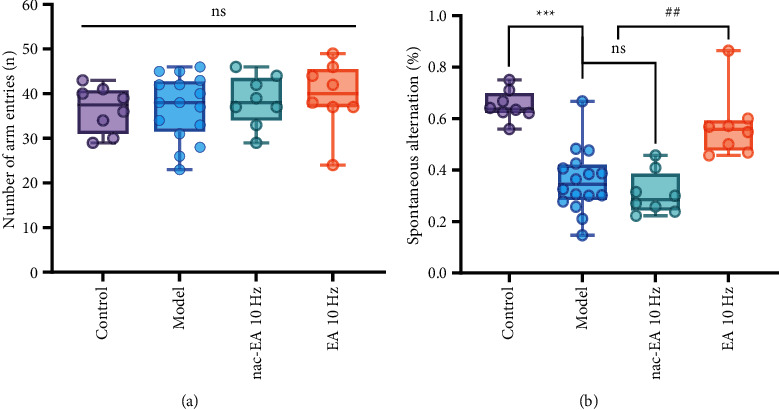
EA at 10 Hz improves the working memory of SAMP8 mice, as measured by the Y maze test. (a) The histogram shows that there was no significant difference in the total number of arm entries among the 4 groups. (b) The histogram shows that the correct spontaneous alternation rate was low in the model group and was significantly increased in the 10 Hz EA group (*n* = 8/16; compared with the control group, ^∗∗∗^*P* < 0.001; compared with the model group, ^###^*P* < 0.001; ns: not significant).

The Y maze spontaneous alternation test was used to examine the spatial working memory of the mice. As shown in Figure 2(a), there was no significant difference in the total number of arm entries among the four groups (*P* > 0.05), indicating that aging-induced cognitive impairment and 10 Hz EA did not affect the motor ability of the mice. However, the correct spontaneous alternation rate in the model group was significantly lower than that in the control group (*P* < 0.001), whereas 10 Hz EA treatment increased the correct spontaneous alternation rate of SAMP8 mice (*P* < 0.001; Figure 2(b)). nac-EA at 10 Hz did not increase the correct spontaneous alternation rate of SAMP8 mice. These results suggest that 10 Hz EA treatment can improve the working memory of SAMP8 mice without affecting their motor ability.

### 3.2. EA at 10 Hz Rescues Learning and Memory Deficits in SAMP8 Mice, as Evaluated by the MWM Test

**Figure 3:**
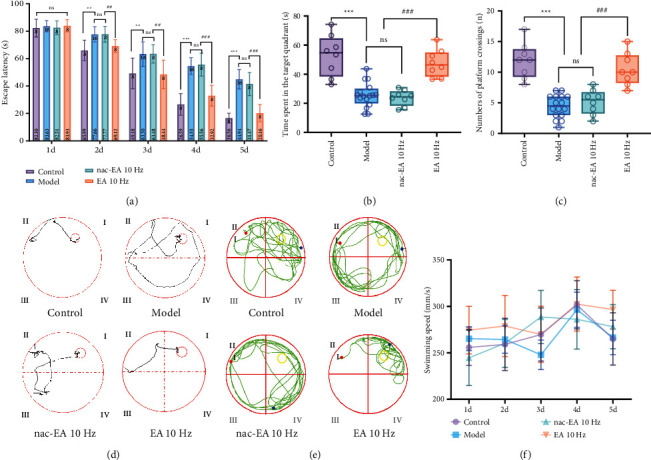
EA at 10 Hz rescues spatial learning and memory impairment in SAMP8 mice. (a) The histogram shows the average latency of the mice to reach the hidden platform on the 5 consecutive training days. (b) The histogram shows the percentage of time the mice spent in the target quadrant during the probe test. (c) The histogram shows the number of hidden platform location crossings. (d, e) Representative swimming tracks of the mice in the test phase on day 5 of the test and the probe test on day 6. The large circle represents the water maze, and the small circle represents the platform. (f) There was no significant difference in the average swimming speed among the four groups of mice over a 5-day period (*n* = 8/16; compared with the control group, ^∗∗^*P* < 0.01 and ^∗∗∗^*P* < 0.001; compared with the model group, ^##^*P* < 0.01 and ^###^*P* < 0.001; ns: not significant).

Figures 3(a)–3(e) show that mice in the control group quickly reached the platform from the entry point after 5 days of continuous training, showing a short swimming distance and strong purpose. After entering the water, mice in the model group showed a random swim path, little differences in the time spent and distance traveled in each area, and a significantly lower search efficiency than those of the control group, indicating that the cognition of the mice in the model group was impaired. However, the movement paths of the mice in the 10 Hz EA group were significantly shortened, indicating that 10 Hz EA improved the cognitive function of SAMP8 mice to some extent. Moreover, compared with mice in the control group, mice in the model group showed an increase in escape latency (*P* < 0.01) and significant decreases in the percentage of time spent in the target quadrant (*P* < 0.01) and number of platform crossings (*P* < 0.01). After 10 Hz EA treatment, the escape latency of the mice (*P* < 0.01) was decreased and the percentage of time spent in the target quadrant (*P* < 0.01) and number of platform crossings (*P* < 0.01) were increased, indicating that the cognitive function of mice in the 10 Hz EA group was significantly improved. There was no significant difference in these measures between the 10 Hz nac-EA group and model group (*P* > 0.05). In addition, as shown in Figure 1(f), there was no significant difference in the average movement speed among the four groups (*P* > 0.05), indicating that the differences reported above were not related to the motor function of the mice. These results suggest that 10 Hz EA treatment rescues spatial learning and memory impairment in SAMP8 mice without affecting vision or motor ability.

### 3.3. EA at 10 Hz Reduces the Level of OxA in the CSF of SAMP8 Mice

**Figure 4:**
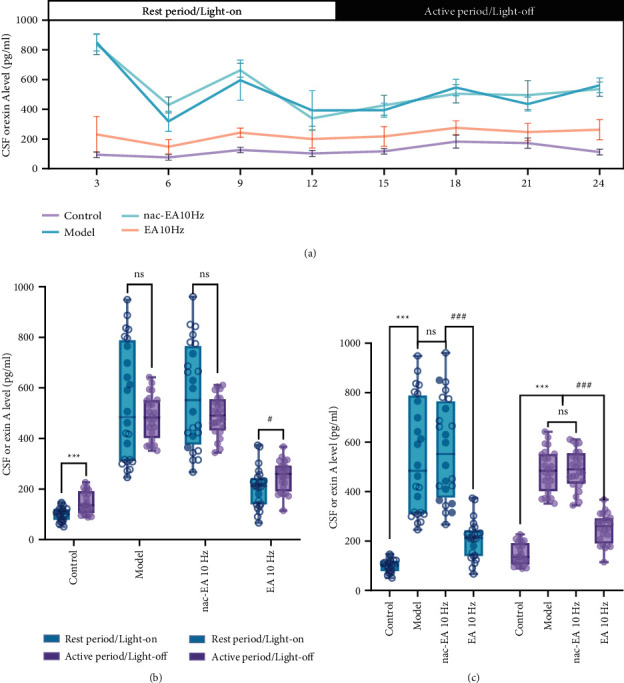
EA at 10 Hz reduces the 24-hour level of OxA in the CSF of SAMP8 mice. (a) The effect of 10 Hz EA treatment on the 24-hour CSF OxA concentration. (b) Comparison of the mean concentration of OxA in the CSF of SAMP8 and SAMR1 mice during the light and dark periods (compared with the rest period (light period), ^∗∗∗^*P* < 0.001 and ^#^*P* < 0.05; ns: not significant). (c) The mean concentration of OxA in the CSF of SAMP8 and SAMR1 mice in the light and dark periods was compared (*n* = 6; compared with the control group, ^∗∗∗^*P* < 0.001; compared with the 10 Hz EA group, ^###^*P* < 0.001; ns: not significant).

The OxA level in the CSF of SAMR1 mice was the lowest in the middle of the light phase (ZT6) and the highest in the middle of the dark phase (ZT18). In contrast, the concentration of OxA in SAMP8 mice was the lowest during the middle of the light phase (ZT6) and the highest during the light phase (ZT3) (Figure 4(a)). The average OxA content in the CSF in both the light and dark periods was calculated (Figures 4(b) and 4(c)), and it was found that the average OxA level in SAMP8 mice was significantly higher than that in SAMR1 mice throughout the light and dark periods (*P* < 0.001). The concentration of OxA in the CSF of SAMP8 mice decreased immediately after 10 Hz EA and was significantly decreased at all 8 time points after the treatment (Figure 4).

### 3.4. EA at 10 Hz Reduces the Level of Glutamate in the CSF of SAMP8 Mice

**Figure 5:**
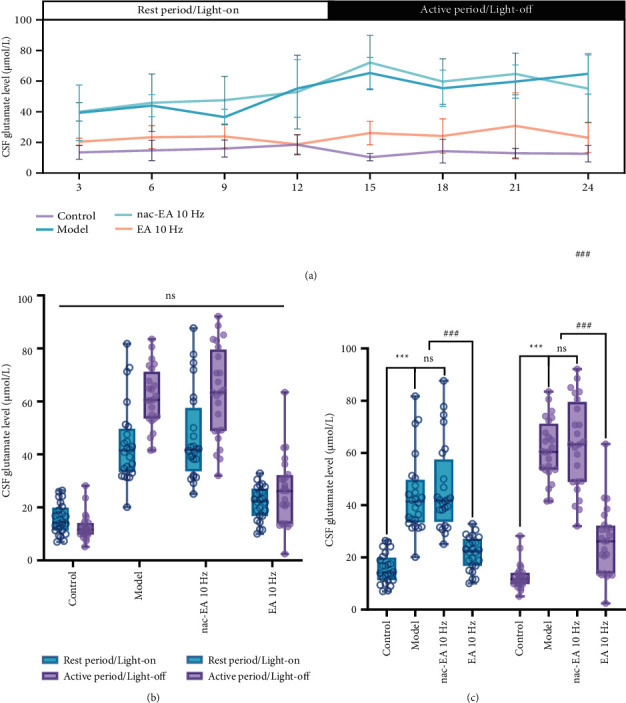
EA at 10 Hz reduces the level of glutamate in the CSF of SAMP8 mice at 24 h. (a) The effect of 10 Hz EA treatment on the 24 hour concentration of glutamate (Glu) in the CSF. (b) The average concentration of glutamate in the CSF in the light and dark phases was compared between SAMP8 and SAMR1 mice (ns: not significant). (c) Comparison of the mean concentration of OxA in the CSF in the light and dark phases between SAMP8 and SAMR1 mice (*n* = 6; compared with the control group, ^∗∗∗^*P* < 0.001; compared with the model group, ^###^*P* < 0.001; ns: not significant).

In contrast to the diurnal changes in OxA levels, the glutamate content in the CSF of SAMP8 mice did not change significantly throughout the day. However, the glutamate level in the CSF of SAMR1 mice was the highest at the end of the light phase (ZT9) and the lowest at the beginning of the dark phase (ZT15). The glutamate concentration in SAMP8 mice was the highest in the early stage of the dark phase (ZT15) and the lowest in the middle stage of the light phase (ZT9). Thus, the changes in glutamate content between the mice of the two genotypes were the opposite (Figure 5(a)). The average glutamate content in the CSF in the light and dark phases was significantly higher in SAMP8 mice than in SAMR1 (*P* < 0.001) (Figures 5(b) and 5(c)). The glutamate concentration in the CSF of SAMP8 mice decreased immediately after 10 Hz EA and was significantly decreased at all 8 time points after the treatment (Figure 5).

### 3.5. EA at 10 Hz Treatment Reduces the Protein Expression of OxA in the Hippocampus and Lateral Hypothalamus of SAMP8 Mice

**Figure 6:**
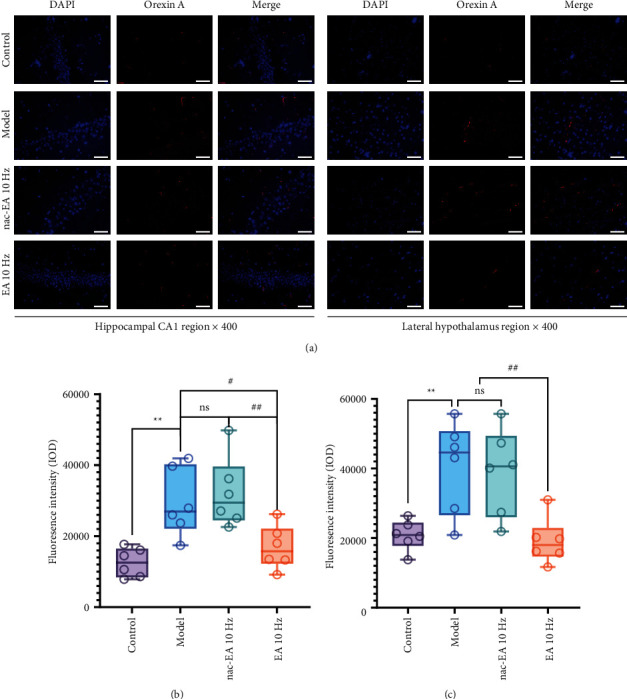
OxA protein expression in the hippocampal CA1 region and lateral hypothalamus of SAMP8 mice was reduced by 10 Hz EA. (a) Representative images of immunofluorescence staining of the OxA protein in the hippocampus and lateral hypothalamus in the 4 groups of mice. (b, c) The histogram shows the integrated optical densities of OxA in the hippocampus and lateral hypothalamus (*n* = 6; compared with the control group, ^∗∗^*P* < 0.01; compared with the 10 Hz EA group, ^##^*P* < 0.01 and ^#^*P* < 0.05; ns: not significant. Magnification, 400x. The scale bar in each figure is 50 *μ*m.

We assessed the distribution of the OxA protein in the hippocampus and lateral hypothalamus of mice by immunofluorescence. As shown in Figure 6, no significant OxA protein expression was detected in the hippocampus or lateral hypothalamus in the control group and 10 Hz EA group, while the OxA protein was significantly expressed in the hippocampus and lateral hypothalamus in the model group and the 10 Hz nac-EA group. The integrated optical densities of OxA in the hippocampus (*P* < 0.001) and the lateral hypothalamus (*P* < 0.001) were significantly decreased.

### 3.6. EA at 10 Hz Treatment Enhances Hippocampal Metabolism and Protects the Neuronal Structure in SAMP8 Mice

**Figure 7:**
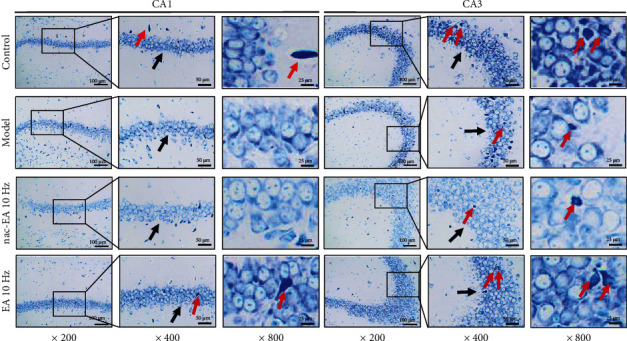
EA at 10 Hz improves the structure of hippocampal CA1 and CA3 neurons and enhances the metabolism of Nissl bodies in SAMP8 mice. (*n* = 3; magnification: 400x, 800x, and 1600x. Scale bars: 50 *μ*m, 25 *μ*m, and 12.5 *μ*m.)

The toluidine blue staining results presented in Figure 7 show that pyramidal cells in the CA1 and CA3 regions of the hippocampus exhibited normal morphology and an ordered arrangement (black arrow) in the control group. Moreover, there were many Nissl bodies, which were visualized as grainy blue-purple bodies (red arrows), among the neurons. In the model group, pyramidal cells in the hippocampal CA1 and CA3 regions were sparse and disordered (black arrow) and Nissl bodies were light in color, sparse in number, loosely distributed, and without hierarchy (red arrow). In the 10 Hz EA group, the pyramidal cells in the hippocampal CA1 and CA3 regions exhibited normal morphology, were orderly arranged, and showed a clear hierarchy (black arrow) and Nissl bodies were darkly stained (red arrow). In the 10 Hz nac-EA group, the arrangement of pyramidal cells in the CA1 and CA3 regions of the hippocampus and the appearance of Nissl bodies were basically the same as those in the model group. This finding suggests that 10 Hz EA can increase the number of Nissl bodies and protect neurons.

### 3.7. EA at 10 Hz Enhances Neuroplasticity by Regulating the OxA-Mediated cAMP/PKA/CREB Signaling Pathway

#### 3.7.1. Effect of 10 Hz EA Treatment on cAMP/PKA/CREB Signaling Pathway-Related Protein Expression in SAMP8 Mice

**Figure 8:**
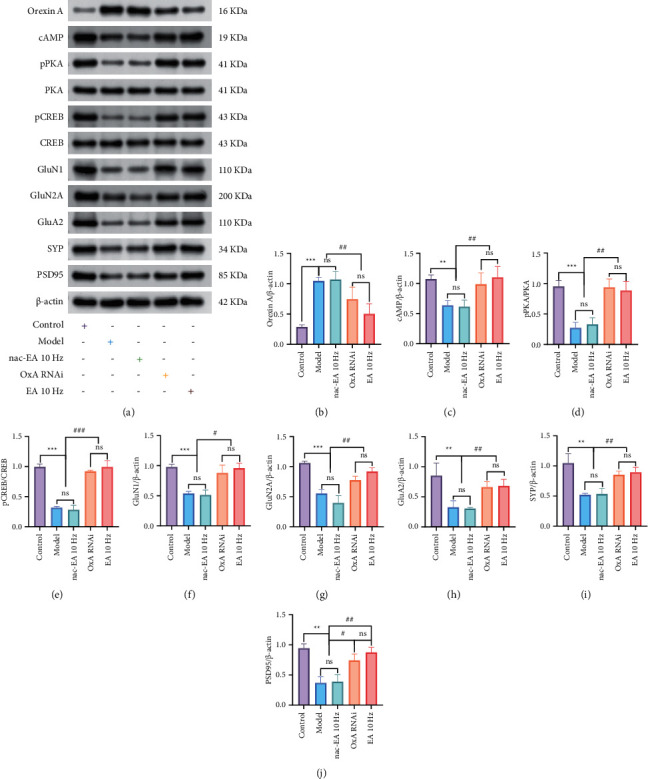
EA at 10 Hz regulates the expression of 9 cAMP/PKA/CREB signaling pathway-related proteins in the hippocampus. (a–j) Western blot images and quantification of the protein levels of OxA, cAMP, pPKA/PKA, pCREB/CREB, GluN1, GluN2A, GluA2, SYP, and PSD95 in the hippocampus. The data are presented as the mean ± standard error of the mean (*n* = 3; compared with the model group, ^∗∗∗^*P* < 0.001 and ^∗∗^*P* < 0.01; compared with the OxA group or 10 Hz EA group, ^###^*P* < 0.001, ^##^*P* < 0.01, and ^#^*P* < 0.05; ns: not significant).

We analyzed the expression of 9 major proteins in the cAMP/PKA/CREB signaling pathway and found that the protein expression of OxA in the model group was significantly increased compared with that in the control group, while the protein expression of cAMP, phosphorylated PKA (pPKA)/PKA, phosphorylated CREB (pCREB)/CREB, GluN1, GluN2A, GluA2, SYP, and PSD95 was significantly decreased (^∗∗^*P* < 0.01 or ^∗∗∗^*P* < 0.001). Compared with that in the model group, OxA protein expression in the OxA-RNAi and 10 Hz EA groups was significantly decreased (^##^*P* < 0.01) and the expression levels of cAMP, pPKA/PKA, pCREB/CREB, GluN1, GluN2A, GluA2, SYP, and PSD95 were significantly increased (^##^*P* < 0.01, ^###^*P* < 0.001). There were no significant changes between the model and nac-EA 10 Hz group (*P* > 0.05) (Figures8(a)–8(j)).

#### 3.7.2. EA at 10 Hz Improves Synaptic Plasticity in Hippocampal Neurons in SAMP8 Mice

Control SAMR1 mice had abundant hippocampal synapses (marked in turquoise), a clear bilayer mitochondrial membrane structure, and organized mitochondrial cristae (purple box indicated by the arrow). The myelin sheaths were intact and dense (green box indicated by the arrow), synaptic structures were clear and intact, the boundary between the presynaptic and postsynaptic membranes was clear, synaptic vesicles were abundant and evenly distributed, and the gap between synapses was tight (blue box indicated by the arrow). The synaptic membrane was intact and highly dense (yellow box indicated by the arrow).

In SAMP8 mice in the model and nac EA group, the number of synapses in the hippocampus was reduced (marked in turquoise), mitochondria were deformed and swollen, and the mitochondrial cristae were disorganized, broken, or even dissolved (purple box indicated by the arrow). The myelin sheaths were structurally incomplete and disintegrated (green box indicated by the arrow), the synaptic clefts were widened, the number of synaptic vesicles in the anterior membrane was reduced (blue box indicated by the arrow), and the posterior membrane was less dense, dissolved, and incomplete (yellow box indicated by the arrow).

In SAMP8 mice in the OxA-RNAi group, synapses were abundant in the hippocampus (marked in turquoise), the mitochondrial bilayer structure was clear, and the internal cristae was arranged in a relatively orderly manner (purple box indicated by the arrow). The myelin sheaths were intact but showed some degree of dissolution (green box indicated by the arrow), the presynaptic and postsynaptic membranes were clearly demarcated, the synaptic clefts were tight, and there were many synaptic vesicles (blue box indicated by the arrow). The postsynaptic membrane was relatively intact and moderately dense (yellow box indicated by the arrow).

**Figure 9:**
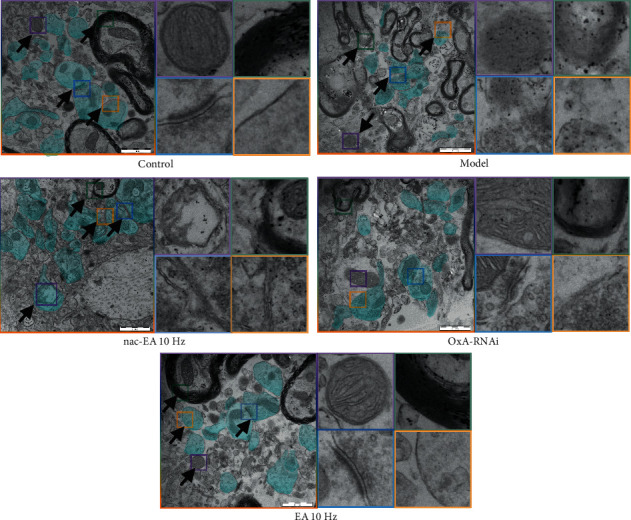
EA at 10 Hz improves hippocampal synaptic plasticity in SAMP8 mice (*n* = 3; scale bar: 1 *μ*m).

In SAMP8 mice in the 10 Hz EA group, the number of synapses in the hippocampus was significantly increased (marked in turquoise), the structure of the mitochondrial bilayer membrane was clear, and the internal cristae was orderly arranged (purple box shown indicated by the arrow). The myelin sheaths were intact, dense, and highly layered (green box indicated by the arrow), synaptic structures were relatively complete, there was a clear boundary between presynaptic and postsynaptic membranes, synaptic vesicles were abundant and uniformly distributed, and synaptic clefts were tight (blue box indicated by the arrow). The synaptic membrane was intact and had a high density (yellow box indicated by the arrow) (Figure 9).

## 4. Discussion

Learning and memory deficits are the main clinical symptoms of dementia. Previous reports have found that various animal models of dementia, including SAMP8 mice, exhibit cognitive and behavioral impairments characterized by impaired learning and memory [22–24]. In this study, 7-month-old SAMP8 mice showed a significant decrease in the percentage of spontaneous alternations in the Y maze test and an increase in escape latency and a decrease in the percentage of time spent in the target quadrant in the MWM test, consistent with previous reports.

OxA is a recently discovered excitatory neuropeptide that plays an important role in regulating circadian rhythm and triggering and maintaining arousal [25, 26]. Circadian rhythms regulate a variety of physiological events, including learning and memory, and disruption of circadian rhythms is closely associated with cognitive deficits [27]. To date, a large number of clinical studies have found that patients with AD have a higher level of OxA in the CSF, which is associated with reduced Mini-Mental State Exam scores and impaired cognitive performance [13, 28–30]. In animal experiments, intraventricular administration of OxA may increase the waking time and affect the circadian rhythm and cognitive function in Tg2576 AD model mice [31]. Zhou et al. [16] found that suvorexant, a dual OXR antagonist, ameliorates behavioral circadian rhythm disturbances observed in 9-month-old APP/PS1 mice. Due to the high clearance rate of suvorexant in rodents, a relatively high dose of the drug, i.e., 30 mg/kg, which is much higher than the recommended dose, is required to enhance synaptic plasticity and inhibit A*β* production, limiting its clinical use [32, 33]. Therefore, new safe and reliable treatment methods are urgently needed. Our team has been committed to the study of learning and memory disorders for many years. Previous studies have found that EA can effectively reduce the OxA level of insomnia patients after stroke [15]. In this study, we administered 10 Hz EA for 14 days, a treatment strategy that was previously confirmed to be efficacious [19], and found that 10 Hz EA effectively reduced the OxA level in the CSF of 7-month-old SAMP8 mice within 24 h. The spontaneous alternation rate in the Y maze test and the percentage of time spent in the target quadrant in the MWM probe test were increased, and the latency to find the hidden platform in the MWM test was decreased, indicating that the impairment of working memory and long-term spatial memory was effectively alleviated in SAMP8 mice. These results are similar to those of previous reports showing that direct injection of OX1R antagonists into the hippocampus can relieve social learning and memory impairment caused by acute stress in rats [34]. Orexin-1 receptor blockade differentially affects spatial and visual discrimination memory facilitation by intracranial self-stimulation [35].

Eyigor et al. [36] found that glutamate axon terminals are in contact with orexin neurons, as revealed mainly by the presence of vesicular glutamate transporters in the terminals, and that these contacts establish synapses on orexin neurons, as determined by ultrastructural analysis. Glutamate, as a major excitatory amino acid neurotransmitter in the hypothalamus, has been shown to regulate wakefulness and support orexin neurons [37, 38]. Glutamate agonists induce excitatory postsynaptic currents in orexin neurons, and this action can be blocked by specific glutamate receptor antagonists [39]. The glutamate level in the hippocampus is closely related to learning and memory, as neuron metabolism and structure can be disrupted by excitotoxicity [40]. To explore the potential mechanism by which 10 Hz EA treatment improved the cognitive function of SAMP8 mice by reducing OxA levels, we performed 24-hour dynamic microdialysis combined with ELISA to assess the changes in glutamate concentrations in the CSF. EA at 10 Hz effectively reduced glutamate levels in the CSF of 7-month-old SAMP8 mice within 24 h. It also improved the structure of neurons in the hippocampal CA1 and CA3 regions and the metabolism of Nissl bodies. Therefore, whether OxA improves glutamate neuron plasticity has become the main question related to the ability of 10 Hz EA to improve the learning and memory of SAMP8 mice. However, it is worth noting that how OxA affects learning and memory by influencing glutamine-ergic neurons has not yet been confirmed. Therefore, in this study, we silenced the OxA gene in the hippocampus to identify the potential regulatory mechanism.

**Figure 10:**
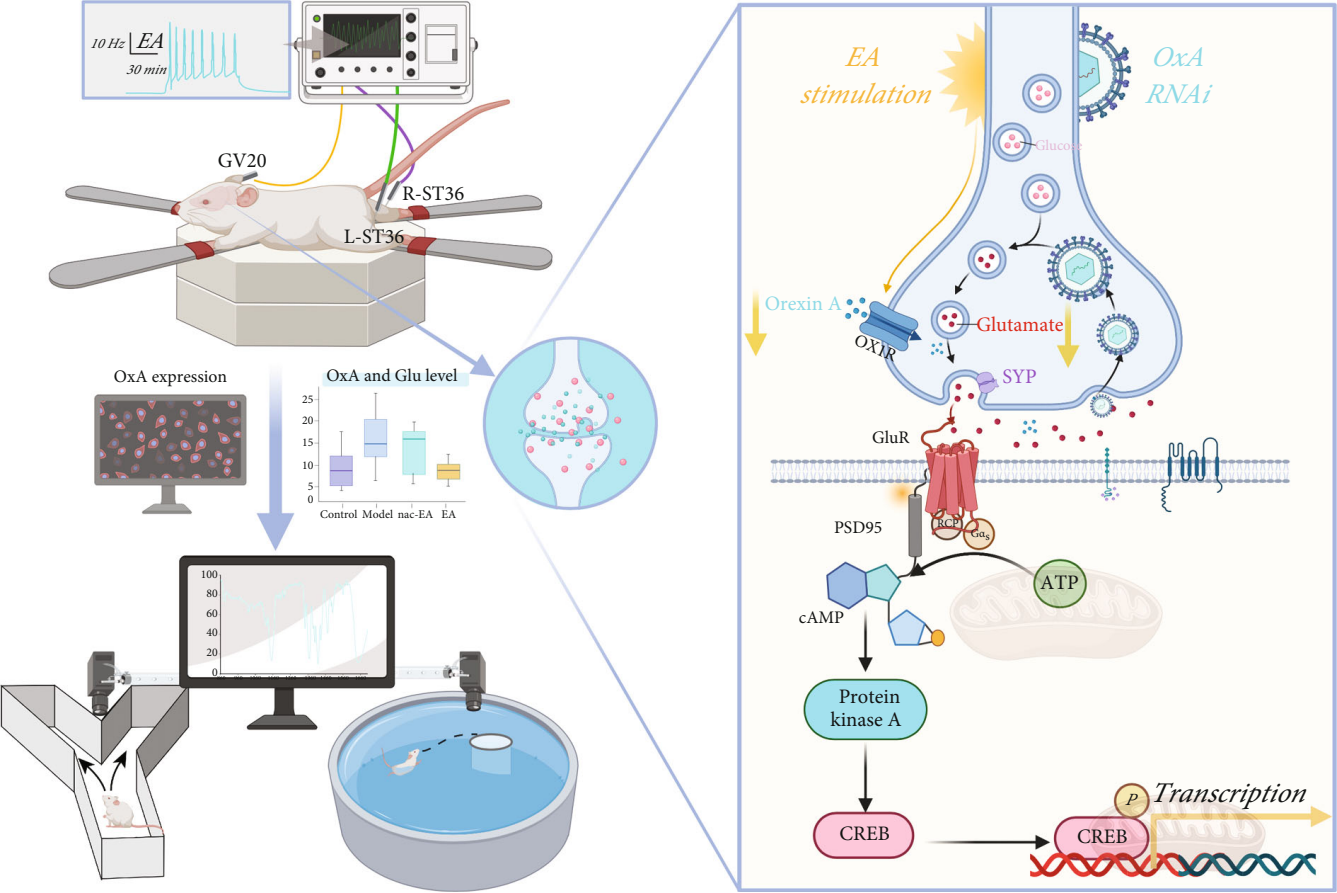
EA at 10 Hz improves hippocampal neuroplasticity in SAMP8 mice to alleviate learning and memory deficits. Inhibition of OxA expression promotes the expression of cAMP/PKA/CREB signaling pathway-related proteins, thus improving the synaptic plasticity of glutamate neurons and reversing learning and memory deficits.

The cAMP/PKA/CREB signaling pathway is closely related to glutamate synaptic plasticity [41, 42]. As an important second messenger, cAMP is involved in the process of learning and memory and changes in long-term synaptic plasticity. Evidence suggests that phosphorylated pCREB in the hippocampus is an activity-dependent transcription factor that plays an important role in synaptic plasticity and long-term memory in SAMP8 mice [43]. It has been found that PKA/CREB is a key upstream factor of NMDAR and that activation of PSD95 promotes pPKA and pCREB expression and thus the formation of new synapses [44, 45]. Our study found that 10 Hz EA has the same effect as OxA-RNAi, i.e., it upregulates the expression of cAMP and promotes the phosphorylation of PKA and CREB, which is consistent with the results of previous studies. It has been found [46] that glutamate is released from presynaptic neurons and transmits signals from presynaptic neurons to postsynaptic neurons through two ionic glutamate receptors, namely, NMDAR and AMPAR, which have various postsynaptic effects. The NMDAR subunits GluN1 and GluN2A, the AMPAR subunit GluA2, SYP on the presynaptic membrane, and PSD95 on the postsynaptic membrane are the major markers of synaptic plasticity in glutamate neurons [47, 48]. In this study, 10 Hz EA treatment and OxA silencing promoted the protein expression of GluN1, GluN2A, GluA2, SYP, and PSD95. The number of synapses in the hippocampus of SAMP8 mice was increased, mitochondrial metabolism was active, and neurotransmission was normal (Figure 10).

The limitations of this study are as follows. First, acupoint specificity is the main factor affecting the efficacy of EA therapy, in both clinical and animal studies. Our previous study found that 10 Hz EA treatment at the GV20 and ST36 acupoints can effectively and consistently induce cell pyroptosis. In this study, we found that the same treatment improved neuroplasticity; however, whether the excitatory neurotransmitter OxA is the main regulator of relevant biological changes needs to be further explored. Second, due to invasive detection methods such as adenovirus injection and microdialysis, we were unable to guarantee the survival of SAMP8 mice in the OxA RNAi group in the preliminary test. Therefore, the formal experimental protocol is not involved in behavioral and other related studies in the OxA RNAi group. Third, since the GV20 acupoint is on the head and the ST36 acupoint is on the legs, the exact pathway by which this nondrug therapy alleviates learning and memory deficits is unclear. The transmission of EA signals delivered at two relatively distance points to the hippocampus affects the translation of proteins related to the cAMP/PKA/CREB signaling pathway. Recently, a landmark study from Q. Ma's team uncovered that [17] electroacupuncture at ST36 points can drive the vagus nerve-adrenal anti-inflammatory pathway and play a role in inhibiting inflammation. A class of PROKR-CRE-labeled DRG sensory neurons was revealed, which laid a neuroanatomical foundation for the existence of ST36 acupoint specificity. However, the neuroanatomical basis of the acupoint specificity of GV20 is still unclear and how the combined treatment of ST36 and GV20 plays a synergistic role in improving cognitive function needs to be further explored.

## 5. Conclusions

In conclusion, this study suggests that 10 Hz EA treatment regulates glutamate synaptic plasticity mediated by cAMP/PKA/CREB by reducing OxA levels in the CSF, ultimately ameliorating cognitive impairment in SAMP8 mice, possibly providing a new option for nondrug therapy for patients with age-induced cognitive impairment.

## Data Availability

The data used to support this research are available from the corresponding author upon request.
